# Transcutaneous vagus nerve stimulation as a potential novel treatment for cyclic vomiting syndrome: a first case report

**DOI:** 10.1007/s10286-023-01002-3

**Published:** 2023-12-19

**Authors:** Angelica Carandina, Costanza Scatà, Ludovico Furlan, Chiara Bellocchi, Eleonora Tobaldini, Nicola Montano

**Affiliations:** 1 Department of Clinical Sciences and Community Health, University of Milan, Milan, Italy; 2 Department of Internal Medicine, Fondazione IRCCS Ca’ Granda Ospedale Maggiore Policlinico, Milan, Italy

**Keywords:** Transcutaneous vagus nerve stimulation, Cyclic vomiting syndrome, Case report, Dysautonomia, Neuromodulation

Cyclic vomiting syndrome (CVS) is a rare disorder characterized by recurrent episodes of intense nausea and vomiting that can last for a few hours up to several days [1]. This disorder is more common in children, with a mean age of onset from 3.5 to 7 years [2]. More recently, an adult form of the disease has also been identified, and associated with significant impairment of social and occupational functionality [3]. Evidence has emerged on the link between CVS and autonomic dysfunction, with orthostatic intolerance, postural orthostatic tachycardia and abnormal skin sympathetic nervous activity observed in the majority of patients with CVS [4–6]. CVS has also often been described as an abdominal variant of migraine [7] since these two disorders share some clinical features (e.g., nausea, photophobia), as well as periodicity and types of triggers (e.g., acute stress and sleep deprivation) [7]. Several patients with CVS report comorbidity and/or family history of migraine [1, 2]. Current knowledge on the pathophysiological mechanisms of CVS is scanty, leading to serious difficulties in identifying an effective treatment. Antiemetic drugs have been often proposed as treatments [1], although these are associated with multiple side effects and rarely accompanied by a complete remission of the episodes.

Here we report our experience with a young adult female patient who presented to our outpatient clinic for CVS-related dysautonomia. Initially, we investigated her autonomic profile to better understand the underlying pathophysiology of her disorder and then we proposed transcutaneous auricular vagus nerve stimulation (tVNS) as a non-pharmacological treatment based on neuromodulation.

The patient was a 20-year-old woman affected by CVS with childhood onset. The diagnosis of CVS was confirmed at our clinic according to the Rome IV criteria [8]:

1. Presence of stereotypical episodes of vomiting regarding onset (acute) and duration (< than 1 week);
2. At least three discrete episodes in the prior year and two episodes in the past 6 months, occurring at least 1 week apart;
3. Absence of vomiting between episodes, but other milder symptoms can be present.

Since the age of 12, the patient had CVS attacks characterized by a prodromal phase of intense nausea lasting about 2 days, followed by 3–4 days of a vomiting phase. The first vomiting episode started about 5 days before her first menstrual period. However, no synchronization of vomiting episodes with menstrual cycle was reported. The episodes occurred periodically every 45–60 days. Typically, the attack began during the night with nausea, followed by several episodes of vomiting, frequent enough to cause anorexia and severe dehydration. The between-episode interval was completely free of symptoms. However, the CVS severely impaired her quality of life, with effects on both work and study ability. The patient took estrogen-progestin pills for 3 years, during which the episodes always occurred every 2 months. A previous gastroscopy showed some esophageal erosions of about 2–3 mm compatible with lesions deriving from the episodes of CVS, but no other abnormalities were revealed. A complete neurological work-up with brain computed tomography, electroencephalogram (ECG) and brain magnetic resonance was negative.

The patient was transferred from the emergency department to our outpatient clinic for further investigations. She provided written informed consent for participation in a clinical trial for the treatment of dysautonomic symptoms with tVNS. The study was approved by the local Ethics Committee (approval number: 441_2022) in accordance with the ethical standards laid down in the 1964 Declaration of Helsinki.

During the study period, the patient did not take medications, and there were no particular changes in terms of daily habits, diet and physical activity.

In order to evaluate a possible dysautonomia, we performed 7-day continuous ECG recordings with a wearable patch monitor (RootiRX; Rooti Labs, Taipei, Taiwan) for 4 consecutive weeks to evaluate the circadian rhythm of cardiovascular autonomic control (CAC) before and during a vomiting episode. The data extracted from the 7-day recordings were related to heart rate (HR), posture and actigraphy sampled minute by minute. The daily HR increase (∆HR) from sleep (supine position) to wakefulness (orthostatism) was obtained as the difference between the mean HR of the 4-h period before awakening and the mean HR of the 4-h period after awakening. The ECG monitoring revealed a severe HR increase (∆HR) in the morning of the days more distant from the vomiting episode (Fig. 1a, b). The mean (± standard deviation [SD]) ∆HR of the first 7 days of monitoring was 55 ± 10 (range: 40–70) beats per minute (bpm). A progressive reduction of ∆HR in the days preceding the vomiting episode was also observed (Fig. 1c), starting from a ∆HR of 60 bpm (1st day) up to 17 and 19 bpm (6th and 7th day respectively). We performed a beat-to-beat analysis, in the same time intervals selected for the HR analysis, to assess the differences in HR variability (HRV) between sleep and wakefulness across the monitoring days. The SD of NN intervals (SDNN) and the root mean square of successive RR interval differences (RMSSD), both indices that quantify the amount of variability in inter-beat interval measurements [9], were drastically reduced from sleep (supine position) to wakefulness (orthostatism) in the morning of the days distant from the vomiting episode (mean ± SD; ΔSDNN: − 52 ± 17 ms; ΔRMSSD: − 24 ± 8 ms). A progressive decrease in the difference of SDNN and RMSSD values recorded during sleep and the values recorded in the morning of the days preceding the vomiting episode was found (mean ± SD; ΔSDNN: − 19 ± 26 ms; ΔRMSSD: − 7 ± 12 ms), similar to observations on the HR trend. Suspecting a possible comorbidity with postural orthostatic tachycardia syndrome (POTS) [10, 11], we performed a head-up tilt test (HUTT) to evaluate the dynamic response of the CAC to passive orthostatism (70°). We continuously recorded the ECG, the respiratory signal and the non-invasive beat-to-beat arterial blood pressure at rest for 10 min, both during a passive orthostatism for 15 min and during the 10-min recovery in supine position. The HUTT revealed an exaggerated HR response during passive orthostatism (∆HR: 38 bpm) in the absence of orthostatic hypotension (mean ∆SBP: − 5.0 mmHg; mean ΔDBP: + 3.5 mmHg, compared to baseline values; Electronic Supplementary Material Fig. 1). The patient reported a slight sensation of palpitation and dizziness during tilt. Therefore, the hypothesis of a comorbidity with POTS was confirmed, according to the diagnostic criteria [11].

**Fig. 1:**
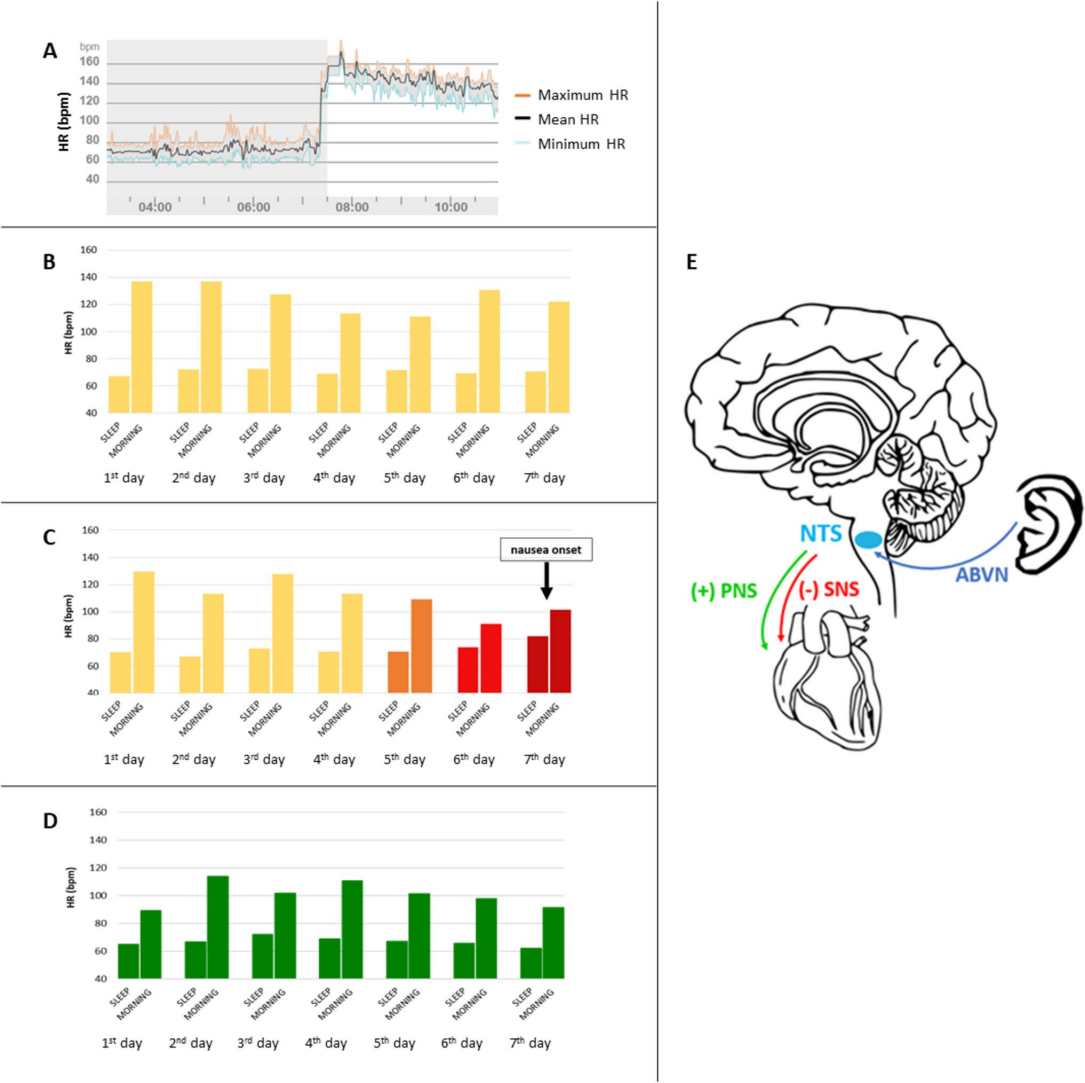
**a** Cardiac response to orthostatic position after awakening: example of HR trend in the hours before and after awakening. **b** Mean HR of the 4 h preceding (sleep) and following (morning) awakening during the first week of monitoring. **c** Mean HR of the 4 h preceding (sleep) and following (morning) awakening in the 7 days immediately preceding the vomiting episode. **d** Mean HR of the 4 h preceding (sleep) and following (morning) awakening after 3 months of tVNS treatment. **e** neuromodulatory pathway activated by tVNS. *ABVN* auricular branch of vagus nerve, *HR* heart rate, *NTS* nucleus tractus solitarius, *PNS* parasympathetic nervous system, *SNS* sympathetic nervous system, *tVNS* transcutaneous auricular vagal stimulation

Both the assessment of CAC and the clinical history of the patient highlighted an impaired autonomic function. Taking into consideration the trend of ∆HR, we hypothesized a slow but constant rise in the hyperactivation of the sympathetic nervous system in the days distant from the vomiting events, followed by an abrupt shift of the autonomic modulation towards an exaggerated vagal activation, ultimately resulting in nausea and vomiting. Given the evidence of a direct involvement of the autonomic nervous system (ANS) and since the CVS shares common features with other neurogenic disorders characterized by neuronal hyperexcitability (e.g. migraine and epilepsy), we proposed to the patient that she start a treatment with transcutaneous auricular vagus nerve stimulation (tVNS), which had been approved as therapeutic approach of drug-resistant epilepsy, chronic migraine and depression [12]. The tVNS was self-administered at home through a portable non-invasive device (VITOS; tVNS Technologies GmbH, Erlangen, Germany). The stimulation parameters included a duty cycle of 30 s on and 30 s off, a pulse width of 250 μs, a frequency of 25 Hz and a biphasic square waveform. The ear electrode was placed in contact with the skin of the left cymba conchae. The stimulation intensity (range: 0.2–5 mA) was set by the patient daily on the basis of personal minimum intensity required to perceive electrical stimulation, described as a pricking or tingling sensation on the skin without discomfort [13]. The treatment compliance was monitored through the device report. The tVNS was performed 4 h per day for 8 months at home. After 1 month of tVNS the patient experienced a new vomiting episode; however, the episode was of mild intensity and shorter (2 days instead of 3–4 days). We repeated the 7-day ECG monitoring after 3 months of tVNS. The after-treatment mean (± SD) ∆HR from sleep to wakefulness was 34 ± 8 (range: 24–47) bpm and it was diminished with respect to the pre-treatment monitoring (Fig. 1d vs. Fig. 1b, respectively). A similar trend was observed for the SDNN and RMSSD indices, with a smaller reduction in variability in the transition from sleep to wakefulness (mean ± SD; ΔSDNN: − 13 ± 10 ms; ΔRMSSD: − 10 ± 7 ms).

The tVNS treatment was then discontinued. Four weeks after the discontinuation of the tVNS, the patient experienced a new episode of nausea and vomiting lasting 4 days. Therefore, the tVNS treatment was resumed for the duration of 5 months and then discontinued again. The latest clinical follow-up has thus far documented a persistent asymptomatic period of 8 months after the second discontinuation of stimulation.

This is the first case report on the efficacy of tVNS on CVS. The present case study provides new evidence on the possible causal relationship between autonomic dysfunction and CVS. The observed beneficial results of tVNS on clinical features of both CVS and POTS could be due to a neuromodulatory effect of this technique. It is known that tVNS directly engages the nucleus tractus solitarius (NTS; Fig. 1e), which is the primary brainstem target of most afferent vagal projections, and significantly activates areas of the central vagal network, namely the locus caeruleus, amygdala and nucleus accumbens [14]. Vagal afferent signaling to the NTS could have led to an indirect reduction of sympathetic hyperactivation [15, 16], resulting in a reduced HR response to the orthostatism, and a consequent prevention of the vagal rebound that leads to the vomiting episodes. Future studies and randomized controlled clinical trials are needed to confirm the positive effects of tVNS on CVS symptoms and on sympathetic modulation. tVNS could therefore represent an innovative neuromodulatory therapeutic approach for CVS and related dysautonomic features.

### Supplementary Information

Below is the link to the electronic supplementary material.

Supplementary file1 (DOCX 17 KB)

## Data Availability

The data presented in this study are available on request from the corresponding author.
