# Supplementary material for: Transcutaneous vagus nerve stimulation as a potential novel treatment for cyclic vomiting syndrome: a first case report
Source: Clin Auton Res. 2023 Dec 19;34(1):209–12. doi: 10.1007/s10286-023-01002-3 (PMC10944428; doi:10.1007/s10286-023-01002-3)
Supplement: Supplementary file 1 — Supplementary file1 (DOCX 17 KB) [file 10286_2023_1002_MOESM1_ESM.docx]

**Supplementary Material**

**Transcutaneous vagus nerve stimulation as a potential novel treatment for cyclic vomiting syndrome: the first case report**

**Supplementary Material _ Figure 1.** Dynamic response of the cardiovascular autonomic control to 70° head-up tilt test. Abbreviations: HR, heart rate; sBP, systolic blood pressure; dBP, diastolic blood pressure.
